# Process Variable Optimization in the Manufacture of Resorcinol–Formaldehyde Gel Materials

**DOI:** 10.3390/gels4020036

**Published:** 2018-04-17

**Authors:** Martin Prostredný, Mohammed G. M. Abduljalil, Paul A. Mulheran, Ashleigh J. Fletcher

**Affiliations:** Department of Chemical and Process Engineering, University of Strathclyde, Glasgow G1 1XJ, UK; martin.prostredny@strath.ac.uk (M.P.); mohammed.abduljalil@strath.ac.uk (M.G.M.A.); paul.mulheran@strath.ac.uk (P.A.M.)

**Keywords:** xerogel, Brunauer-Emmett-Teller theory, Barrett-Joyner-Halenda analysis, temperature, solids content, drying, solvent exchange

## Abstract

Influence of process parameters of resorcinol–formaldehyde xerogel manufacture on final gel structure was studied, including solids content, preparation/drying temperature, solvent exchange, and drying method. Xerogels produced using a range of solids content between 10 and 40 *w*/*v*% show improved textural character up to 30 *w*/*v*% with a subsequent decrease thereafter. Preparation/drying temperature shows a minimal threshold temperature of 55 °C is required to obtain a viable gel structure, with minimal impact on gel properties for further thermal increase. Improving the solvent exchange method by splitting the same amount of acetone used in this phase over the period of solvent exchange, rather than in a single application, shows an increase in total pore volume and average pore diameter, suggesting less shrinkage occurs during drying when using the improved method. Finally, comparing samples dried under vacuum and at ambient pressure, there seems to be less shrinkage when using vacuum drying compared to ambient drying, but these changes are insubstantial. Therefore, of the process parameters investigated, improved solvent exchange seems the most significant, and it is recommended that, economically, gels are produced using a solids content of 20 *w*/*v*% at a minimum temperature of 55 °C, with regular solvent replenishment in the exchange step, followed by ambient drying.

## 1. Introduction

Resorcinol–formaldehyde (RF) aerogels are a family of porous materials, first discovered in 1989 [1] by Pekala, and which have seen extensive application, due to their tailorable textural properties, in a range of applications, including as catalyst supports [2,3,4], in gas storage systems [5,6] and gas separation devices [7,8], in the fabrication of fuel cells [9,10], and as a core component in insulation [11,12]. The control of the porous character of these materials underpins their vast applicability, allowing tailored synthesis in terms of surface area, pore volume and pore size; however, the mechanism by which these gel materials form is not completely understood and there is significant scope for materials and process optimization.

**Figure 1:**
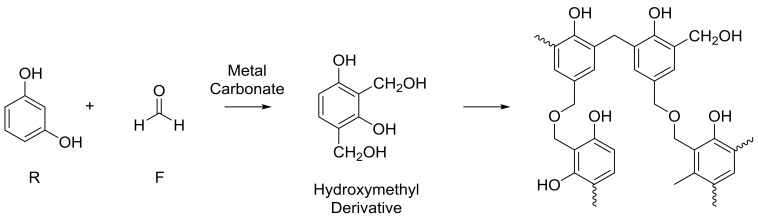
General reaction mechanism proposed in the reaction of resorcinol and formaldehyde. R: resorcinol; F: formaldehyde.

It is generally accepted that the sol-gel polycondensation reaction of resorcinol (R) and formaldehyde (F) proceeds as shown in Figure 1; the reaction is also usually performed at above ambient temperatures. The reaction can be seen to proceed via an initial addition reaction between R and F, forming a hydroxymethyl derivative species, which undergoes self-condensation to create oligomeric chains that form clusters, and finally, a cross-linked 3D gel network. Our previous work, utilizing light scattering techniques, has provided insight into the mechanism of cluster growth, whereby, in a system with fixed reaction parameters, thermodynamics controls the size of growing clusters, while there is kinetic control of cluster population [13]. The reaction is promoted by the presence of a metal salt, known within the field as a catalyst. The most commonly used catalyst is sodium carbonate (Na_2_CO_3_), as originally used by Pekala, and the role of the metal carbonate is thought to be two-fold. While the carbonate is known to act as a base, promoting the initial reaction between resorcinol and formaldehyde through proton abstraction, the metal ion is thought to stabilise the colloidal suspension involved in development of clusters dispersed within the solvent matrix [14]. Hence, many studies have previously focused on the use of different catalytic species to control the final gel material [13,14,15,16,17,18,19]. However, it should be noted that the polycondensation reaction can also be influenced by a variety of other synthesis parameters, resulting in a modification of the porous character of the final aerogel product [20,21]. This includes synthesis parameters such as resorcinol to carbonate molar ratio (R/C) and the mass of solids dissolved within a fixed volume of solvent (deionised water) [22], as well as process variables, which can also affect the end material. Recent research has shown that both the time allowed for the reaction mixture to be stirred before heating [23], and the shape of the mould used to form the RF aerogel [24], can also have a significant effect on the internal structure of the gel product. The post-synthetic processing of RF gels is also subject to significant variation, in terms of solvent exchange and drying methods used, the former is usually selected to enhance the latter. Drying methods include supercritical drying, freeze drying or ambient temperature drying, with or without vacuum.

Kistler was instrumental in developing the first aerogels from silica based materials, and in his work, he had observed that evaporative drying results in destructive forces acting on the pore walls as a consequence of surface tension, and subsequent collapse of the gel [25]; he also established that, due to the high critical temperature and pressure of H_2_O [26], and its poor solubility in supercritical solvents [27], the water entrained within the gel first had to be exchanged with a solvent that was completely miscible with the supercritical solvent. Following this work, the Lawrence Berkeley Laboratory [28] discovered that supercritical CO_2_ could be used as a direct solvent replacement in the drying of silica aerogels [28], presenting a safer route to gel production. Pekala subsequently used this discovery, in conjunction with previous knowledge on RF resins to prepare organic aerogels [1,29]. Further studies, since then, discovered that other drying methods can be used, e.g., conventional evaporative drying to form xerogels [30] and freeze drying to form cryogels [31,32,33,34]. Czakkel et al. [32] compared the effects of evaporative drying in an inert atmosphere, freeze drying and supercritical drying, on the textural properties of RF gels, and found that the cryogels exhibited the highest pore volumes and surface areas due to the improved solvent quality of t-butanol; the evaporative samples showed less developed structures due to increased shrinkage arising from the formation of a liquid–vapour interface and resultant surface tension [20]. This indicates that the final drying step is critical to retention of porous character; however, Jabeen also demonstrated that exchanging entrained water with a solvent of lower surface tension reduced gel shrinkage and, as a result, increased pore volume [35]. The results indicate that, even in the event of a prolonged solvent exchange, residual water is retained within the pores of the gel, resulting in increased surface tension during drying, and impacting on the porous structure obtained. Another way to avoid liquid–vapour interfaces is to use freeze-drying [12,13,14,15]. It has been noted, in previous studies, that supercritical drying and freeze-drying are expensive to perform, and require specialist knowledge [34,36]; hence, a route to gel production that avoids such methods would be economically beneficial.

These previous works have established a base from which most researchers work to produce RF gels but, to date, there has been no overarching study that has investigated process optimization holistically, which is essential for the scaled production of these materials. Hence, the aim of this current work is to optimise synthesis parameters and process variables to provide tailored production of selected textural characteristics in the final material. This involves determination of the impact of the solvent exchange method, total solids content, and drying method used, with respect to with varying R/C ratio. This optimal system was then studied further by altering the temperature at which the steps of synthesis, curing and drying were all set, to determine the validity of the widely accepted temperature of 85 °C in the synthetic procedure, as this has potential impact on the basis of both economics and safety. Low temperature nitrogen sorption measurements were used to characterise the textural properties of the synthesised aerogels, allowing changes in the internal structure of the xerogel to be monitored and quantified.

## 2. Results and Discussion

### 2.1. Effect of Solvent Exchange Method

Gels, produced as outlined above, generally undergo solvent exchange for a period of three days with only an initial volume of acetone added to the drained, cured gel; however, this may not be the most appropriate method to retain the porous structure developed during synthesis. Due to the high surface tension value for water, over the synthetic temperature range used to produce RF gels, the process of drying hydrogels leads to significant shrinkage of the material, as a consequence of the resulting high stresses that act on the pore walls. Therefore, it is desirable to replace the water, entrained within the pores, with a liquid that exhibits a lower surface tension, and preferably a lower boiling point, than water, within the temperature range of interest. The surface tension of water is high, even at elevated temperatures, e.g., 67.94 N/m at 50 °C [37], and a number of alternative solvents, with reduced surface tensions e.g., amyl acetate, acetone, t-butanol and isopropanol [20,38], have been proposed for solvent exchange in previous studies; however, acetone offers an excellent combination of a reduction in surface tension (19.65 N/m at 50 °C [39]) and relatively low cost compared to alternative solvents. Hence, acetone was used for solvent exchange within this study.

Replacement of the liquid within the pores is driven by diffusion, although agitation is often used to enhance contact of the material and fresh solvent; hence, sufficient time is required for full exchange to occur. Another factor that is potentially important, in maximizing the level of exchange, is the water concentration gradient between the pore liquid and the bulk solvent surrounding the sample. To investigate the effect of the solvent exchange method used, three batches of gels, individually of 60 mL total liquid volume, were synthesised, each of which, after curing, were washed with acetone, drained and, subsequently, agitated in acetone for three days. The key difference was that the first two batches were used to investigate the effect of a different volume of acetone used in one application and were processed by adding the entire volume of acetone at the beginning of the three days, namely 180 or 240 mL, and the sample was left without further handling for the whole solvent exchange period, while the third batch was treated with a fresh volume of acetone each day for three successive days with the total volume of acetone used adding up to 240 mL, thus maintaining the same total volume of acetone as the second batch but splitting the total volume over multiple days.

**Figure 2:**
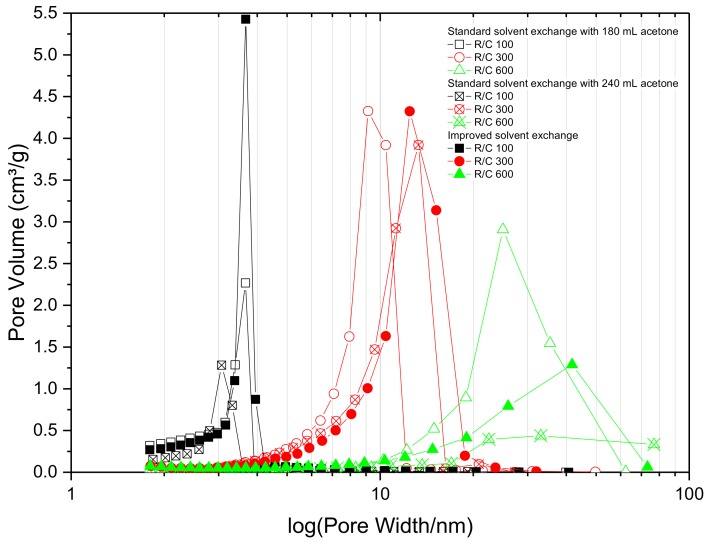
Effect of solvent exchange method on pore size distribution for resorcinol–formaldehyde xerogels with varied resorcinol:carbonate (R/C) molar ratio.

The data obtained for the pore size distributions of the three batches of gels are shown in Figure 2, and it can be seen that changing the acetone bath daily has a more pronounced positive effect on the total pore volume of the RF gel samples compared to just increasing the total acetone volume without changing the bath daily, especially for samples with lower R/C ratios. Improving the solvent exchange method, by increasing the concentration gradient daily, leads to pores with larger average diameter (Table 1). This, coupled with the increase in pore volume, is ascribed to a reduction in shrinkage during the drying stage. If the acetone bath is replaced daily, the water concentration gradient is renewed every day, thus there is an increased driving force, which removes more water from the pores. This leads to lower stresses being exerted on the pore walls during the drying stage, due to the lower surface tension of acetone compared to water. However, for samples with higher R/C ratios exhibiting a weaker gel structure, the improved method does not seem to have the same pronounced positive effect observed for the lower R/C gels with smaller average pore diameter. A possible explanation is that when the acetone bath is exchanged daily, the replenishment step slightly damages the softer structure, resulting in lower values of surface area and pore size. The findings from this section of work suggest that there is significant advantage in using an improved solvent exchange method for most of the samples, hence, all samples in the following sections were prepared using daily replenishment of acetone within the solvent exchange stage, with the intention of maintaining the gel structure as close to the original hydrogel structure as possible, without the need to use cryogenic or supercritical processing steps. It is important to note that, in order to obtain improved gel characteristics, it is not necessary to increase the amount of acetone used during the solvent exchange, rather it is imperative to split this amount over the exchange period.

### 2.2. Effect of Changing Solids Content

There is a tendency within the literature to use solids contents of ~20 *w*/*v*% in the production of RF gels [13,14,40,41]; however, the amount of solid material within the reaction volume would be expected to affect the solid:liquid ratio, hence, the final gel characteristics. Here, RF gels were synthesised over the range of solids content between 10 and 40 *w*/*v*%, using R/C ratios of 100, 300 and 600. Note that these samples were prepared at 85 °C, using improved solvent exchange (see Section 2.1) and vacuum drying (see Section 2.3). For the samples synthesised using a solids content of 10 *w/v*%, gelation was unsuccessful for R/C ratios greater than 600, hence, the range used in this study, but it should be noted that R/C ratio can be increased as the solids content increases but would not allow a direct comparison within this work, thus R/C 600 was the highest value studied here. For solids contents ≥20 *w*/*v*%, some of the samples exhibited cracking during the drying stage, which affected their final characteristics.

From Table 2, it can be observed that, at constant R/C molar ratio, there is no significant change in specific surface area as mass content changes; however, the total pore volume is seen to increase with solids content, up to ≤30 *w*/*v*%, after which point, the pore volume is slightly reduced at low R/C but still increases at higher R/C values. This can be ascribed to interplay between R/C ratio, i.e., particle nucleation number, and solids content, i.e., available material for particle growth; this means that the higher R/C ratios are more greatly affected by the additional mass available, due to the lower number of particles formed. The decrease at low R/C may be attributable to inhomogeneity during the gelation process, when no active agitation is applied, or possibly due to the increased mass per unit volume, which increases the relative density and reduces the void space available. Similarly, at constant R/C molar ratio, the average pore size increases with increasing solids content, again to 30 *w*/*v*%, whereupon it decreases steadily with increasing reactant concentration. Increasing the mass of reactants at a fixed R/C ratio, increases both the monomer concentration and that of sodium carbonate, as the catalyst, which leads to an increase in the number of particles formed during gelation; this could result in the observed decrease in average pore size. It should be noted that the pore diameters determined for R/C 100 are constant at three nanometers; however, differentiation at this level is hindered by the size of the probe molecule, which only allows integer values to be reported.

**Figure 3:**
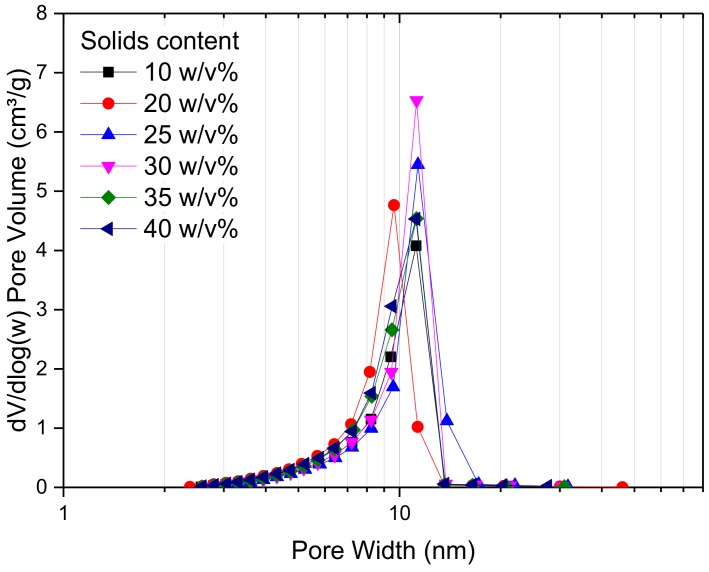
Pore size distribution obtained for resorcinol–formaldehyde xerogels synthesised using a resorcinol:cataylst molar ratio of 300 and varied percentage solids contents.

**Figure 4:**
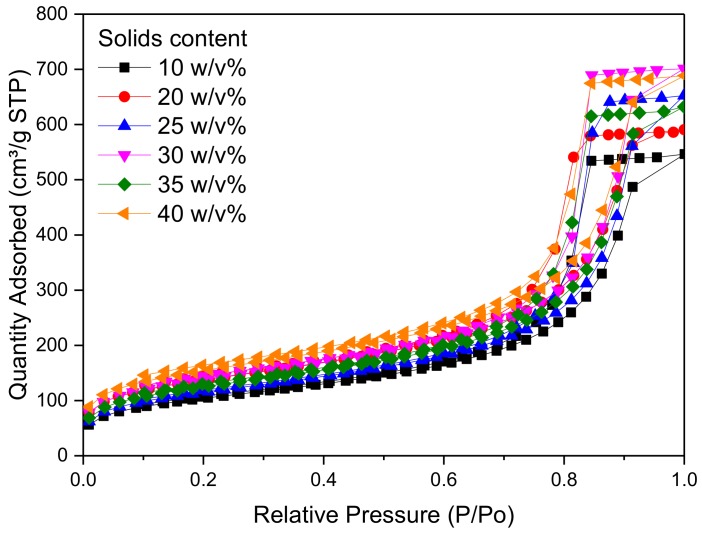
Nitrogen adsorption-desorption isotherms obtained for resorcinol–formaldehyde xerogels using a resorcinol:cataylst molar ratio of 300 and varied percentage solids content.

Figure 3 shows the pore size distribution of RF gel samples prepared at a constant R/C molar ratio of 300, and using different percentage solids contents. It can be seen that there is no significant change in the pore size distribution as the reactant concentration changes; however, it can be observed that RF gels with solids contents of 25 and 30 *w*/*v*% exhibit the narrowest distribution, with a sharp peak at ~15 nm. From Figure 4, meanwhile, it is obvious that altering the solids content has no major effect on overall shape of the adsorption–desorption isotherm of N_2_, with all samples exhibiting Type IV isotherms [42]. The quantity of N_2_ adsorbed increases with increasing relative pressure and a solids content of 30 *w*/*v*% shows the highest adsorption capacity of all levels tested. The combination of a discrete pore size distribution and high pore volume (Table 2) indicates that the selection of 20 *w*/*v*% in the synthetic matrix is in line with process optimization.

### 2.3. Ambient Pressure vs. Vacuum Drying

The final stage of gel preparation is drying of solvent exchanged gels, which, in this case, involves subcritical drying of the gels to remove acetone. The gels prepared in this way exhibit a higher degree of shrinkage; however, it is much easier to implement, and more economical, when compared to supercritical drying with CO_2_. Usually, in order to make the drying process faster, and to ensure that the final materials have been dried thoroughly, vacuum drying is utilised. Maintaining a vacuum during drying is also not inexpensive, so it would be beneficial if RF gels could be dried under ambient pressure at elevated temperature, while retaining their final properties. Therefore, a series of gel samples were prepared, where the gel sample was divided in two halves post improved solvent exchange. This ensured that any effects observed within the final structure only resulted from the selected drying procedure. One half of the sample was dried for two days under vacuum at 85 °C, while the other half was dried under ambient pressure at 85 °C for one day and subsequently moved to the vacuum oven with the other sample half for one day of further drying, this time sub-atmospherically. Most of the drying process occurs during the first day; while the second day is used to remove the final traces of acetone remaining in the pores.

Table 3 shows the textural properties obtained for the gels prepared as outlined above. It can be observed that even though the gels dried under vacuum tend to have higher surface areas, pore volumes, micropore volumes, and larger average pore widths, the differences are insubstantial. This means that, if the requirements for the final material are not too strict, it should be possible to initially dry RF gels at ambient pressure, potentially even in the same oven as is used for gelation, since the temperatures are equivalent. From an industrial perspective, this could result in significant cost savings associated with the drying process of RF gels, and the handling of materials between unit operations, and could make such materials potentially cost-effective for new applications.

### 2.4. Influence of Synthetic and Processing Temperature

In light of the three previous steps, it seems reasonable that the preparation of gels using 20 *w*/*v*% solids content, with an improved solvent exchange step and either ambient or vacuum drying should yield reasonably optimal materials. The constraint of several process variables also indicates that it should be possible to obtain materials with a high degree of reproducibility; however, this is dependent on control of one significant parameter, which can have significant impact on the overall process costs, i.e., temperature. The first stages of resorcinol–formaldehyde (RF) gel formation, immediately after mixing the components, are gelation and curing, which are usually carried out at elevated temperatures, and the final processing steps of gel production also include the use of a raised temperature during drying. Hence, the final parameter studied here was the influence of temperature within the manufacturing process. In all previous experiments, 85 °C was selected as the gelation and curing temperature as gels previously obtained at this temperature have exhibited a viable structure, and it is also a commonly used value in the literature, allowing further comparisons to be made to previously reported results [20,43,44]. It has, however, been shown that RF cluster particles begin to grow once the solution reaches a temperature of at least 55 °C [13], which indicates a minimum threshold for investigation; since water is used as the solvent, in the synthesis outlined above, the upper temperature limit is, therefore, set by the boiling point of water. Thus, the chosen temperature range studied was 45–95 °C, with 10 °C intervals. This allowed the region both above and below the temperature necessary for cluster growth to be probed to determine whether a viable gel structure can be established and maintained at temperatures approaching both (i) the boiling point of water and (ii) lower, less energy demanding, temperatures. R/C ratio was varied, as required, but all other synthesis parameters were kept constant as stated above; the only other change was that of oven temperature during the gelation and drying stages. Due to the enhanced performance observed above, improved solvent exchange was used exclusively, and the drying temperature, used in the vacuum stage, matched the gelation and curing temperatures, in order to restrict any post gelation changes in structure caused by exposure to a higher temperature during drying.

**Figure 5:**
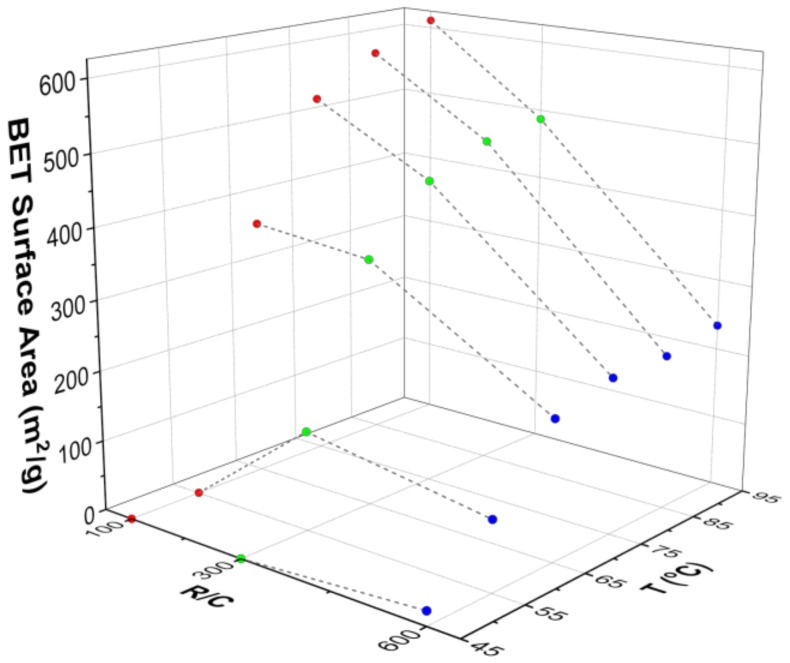
Dependence of BET surface area on resorcinol–formaldehyde xerogel preparation temperature and resorcinol:catalyst (R/C) molar ratio.

Table 4 shows the textural properties for gels synthesised at different temperatures, obtained from nitrogen adsorption analysis. Gels prepared at lower temperatures either did not gel or exhibited a very weak structure that did not withstand the drying process; this led to materials with a low degree of porosity or even to non-porous materials. The effect of temperature can be seen more clearly in Figure 5, where the influence of gel preparation temperature, and R/C ratio, on Brunauer-Emmett-Teller (BET) surface area is shown. It can be seen that, at low temperatures (45 and 55 °C), the surface areas obtained are very low, and are essentially independent of the R/C ratio used. At higher temperatures, the BET surface area seems to be only slightly dependent on temperature, and the effect of catalyst concentration dominates as the major factor in determining the final gel structure properties. These results are in disagreement with results from Tamon and Ishizaka [45] who reported that gelation temperature had no influence on the final gel structure. The difference is likely ascribed to the fact that their gelation step at either 25 or 50 °C was followed by a curing period of five days at 90 °C. Thus, the influence of the lower temperature gelation stage would have been masked by subsequent exposure to the same higher temperature during the curing stage.

**Figure 6:**
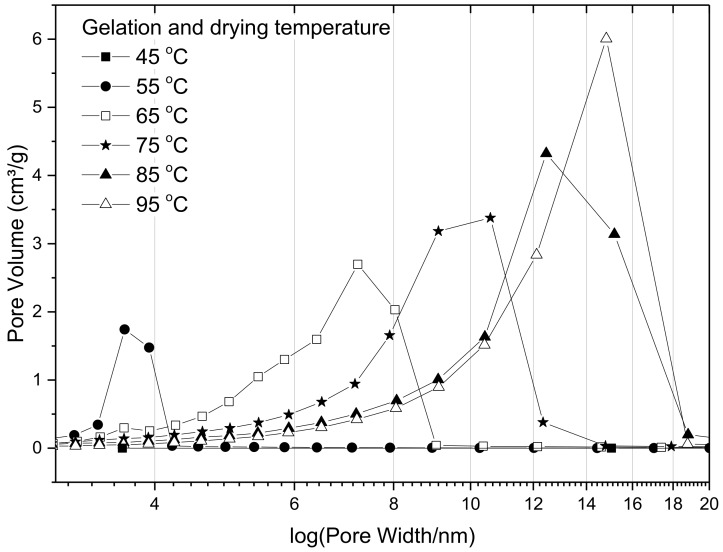
Effect of gelation temperature on pore size distributions for resorcinol–formaldehyde xerogels prepared using resorcinol:catalyst molar ratio of 300 and 20 *w*/*v*% solids content.

Pore size distributions for the suites of samples prepared using different temperatures, and R/C ratio 300, are presented in Figure 6, and the results show that the pore size distribution shifts towards larger pore diameters with increasing gelation temperature. This implies that gels prepared at higher temperatures develop stronger crosslinkages, which leads to a lower degree of shrinkage during the drying stage. It can also be observed that the total pore volume, which is given by the area under the pore size distribution curves, increases with increasing temperature, further supporting the theory that shrinkage is reduced within the stronger structures created at higher temperatures. The gels prepared at 45 °C exhibited such low porosity that the values are not even discernible in Figure 6, and are overlapped by other points; specific values are presented in Table 4.

**Figure 7:**
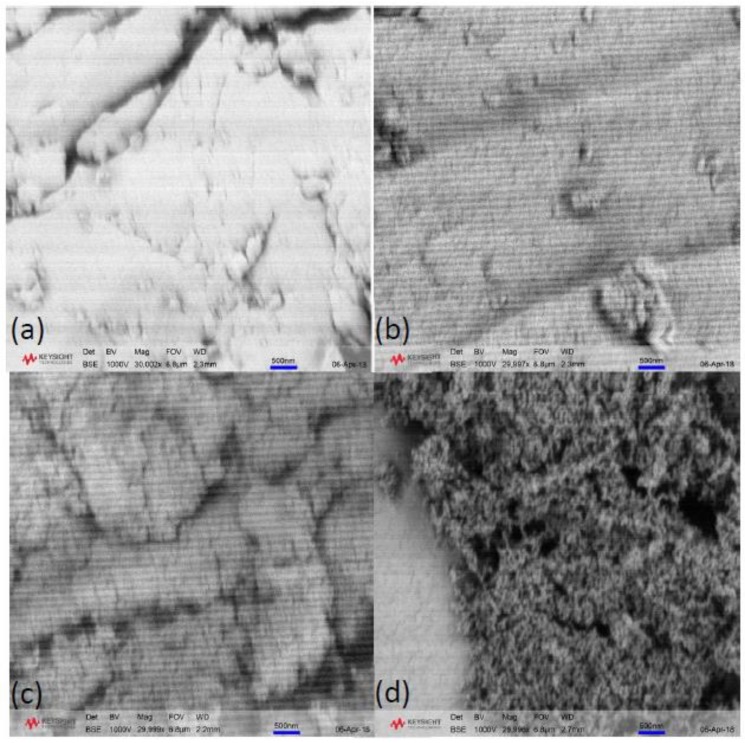
SEM micrographs of resorcinol–formaldehyde xerogels prepared at (**a**) 45 °C with resorcinol:catalyst molar ratio of 100, (**b**) 85 °C with resorcinol:catalyst molar ratio of 100, (**c**) 45 °C with resorcinol:catalyst molar ratio of 600, and (**d**) 85 °C with resorcinol:catalyst molar ratio of 600 at 30,000× magnification.

Morphological images of xerogel samples synthesised at 45 and 85 °C, with R/C ratios 100 and 600, are shown in Figure 7. It can be observed that the samples prepared with R/C ratio 100 do not show any significant textural features at this macroscopic level, which is expected considering the results from nitrogen sorption measurements. The pore size for these samples is below the limit at this magnification and due to the porous nature of the samples, it was not possible to achieve higher magnifications without using a higher thickness of gold coating, which would obscure any fine textural features. By contrast, there is a clear difference in morphology between the samples prepared with R/C 600 at different temperatures. The xerogel prepared at 85 °C (Figure 7d) exhibits a typical porous structure, composed of RF clusters crosslinked into a 3D network with some of the macropores clearly visible. While there are visible differences between samples prepared at 85 °C (Figure 7b,d), the xerogels prepared at 45 °C (Figure 7a,c) exhibit a very similar structure independent of catalyst amount. This agrees with the textural data obtained from nitrogen sorption measurements.

It is evident from these results that, in order to obtain a viable gel structure capable of enduring the drying process, the gelation temperature must be in excess of 55 °C, as suggested by Taylor et al. [13]; however, increasing the temperature further does not seem to have a significant impact on the surface area obtained. The other textural variables are affected slightly and it may be required to use elevated temperatures to optimise a particular variable or enhance the crosslinking within the final gel. This information could be used in process optimization of RF gel manufacture to reduce the heating costs associated with the gelation and drying processes for a specific set of required textural characteristics, as defined by a selected application.

## 3. Conclusions

The work presented here demonstrates the need to carefully control the synthesis and process parameters used in RF gel production, in order to obtain the optimal material for a given application. Solids content is integral to gel viability, with low solids contents resulting in weaker structures that fail to gel at higher R/C ratios, and very high solids contents resulting in increased densification of the material and a reduction in porosity. It was observed that 30 *w*/*v*% represents an upper bound for solids content in the systems studied here, and such materials exhibited the highest accessible pore volume; however, surface area was unaffected by increased mass, at constant R/C. It is suggested that the increased mass of reactants (both monomer and catalyst) increased particle number and decreased average pore size. Within the systems studied, those gels created using solids contents of 20–30 *w*/*v*% exhibited the narrowest distribution; thus, the combination of discrete pore size distribution and high pore volume, with lower reactant costs, indicates 20 *w*/*v*% is optimal for gel production. In line with previous studies, a minimum temperature of 55 °C was shown to be critical in viable gel formation; gels prepared at lower temperatures either did not gel or exhibited a very weak structure with low or negligible porosity, independent of R/C. Gels prepared at higher temperatures showed insignificant changes in surface area with temperature, with the effect of catalyst concentration dominating gel formation; while pore diameter increases with increasing gelation temperature, due to stronger crosslinkages, hence, a lower degree of shrinkage during processing. This indicates that, while the gelation temperature must be in excess of 55 °C, increasing the temperature further has little impact on the final surface area, allowing a lower temperature to be used for gel synthesis if this is a key measure of gel performance. Post-synthesis, the regular replacement of the solvent exchange fluid has a marked positive effect on total pore volume, leading to pores with larger average diameters, which is ascribed to a reduction in shrinkage during the drying stage, due to the increased driving force for water removal, hence, lower stresses being exerted on the pore walls during processing. It is, therefore, not necessary to increase the amount of solvent used within the exchange but it is imperative to increase the number of solvent changes over the exchange period. Finally, the differences between gels dried at atmospheric and sub-atmospheric pressure show little difference in their textural character, hence, it may be possible to dry RF gels at ambient pressure, potentially even in the same oven as gelation, to reduce both heating and pump costs. Combined, these results provide guidance to reduce the costs of RF gel manufacture, without impinging on the desired qualities of the materials produced.

## 4. Materials and Methods

### 4.1. Sample Preparation

Unless otherwise stated, all resorcinol–formaldehyde (RF) gel samples were prepared using an analogous procedure, excepting for the specific parameter investigated in each section of the study. All chemicals were used as received from the supplying company, and deionised water was produced in-house (Millipore Elix^®^ 5 with Progard^®^ 2 (Merck, Watford, UK)). Firstly, the appropriate amount of resorcinol (Sigma Aldrich, Gillingham, UK, ReagentPlus, 99%) was added to a premeasured volume of deionized water in a jar containing a magnetic stirrer bar. Upon dissolution of all of the added resorcinol, a corresponding amount of sodium carbonate (Sigma Aldrich, anhydrous, ≥99.5%), on a molar basis, was weighed out and added to the solution. As outlined above, sodium carbonate acts as a catalyst, by a combination of increasing the solution pH in the basic region via hydrolysis of the carbonate ion, and by the introduction of sodium ions, which, it has been suggested, assist in the addition of formaldehyde to resorcinol [46]. Catalyst concentration is expressed as resorcinol/catalyst molar ratio (R/C) and the range studied here is R/C 100–600. After all solids were dissolved, the required amount of formaldehyde, in the form of formalin solution (Sigma Aldrich, 37 wt % formaldehyde in water, containing 10–15 wt % methanol as a polymerization inhibitor), was added, and the solution was stirred in a closed jar for 30 min. All samples were prepared with 20 *w*/*v*% solids content, unless otherwise stated, and the total volume used was 60 mL, made up of water and methanol, contributed by the formalin solution used. At the end of the period of agitation, stirrer bars were removed from the solution, and the jar lid was hand-tightened, before moving the jar to an oven (Memmert UFE400, Schwabach, Germany) preheated to 85 °C, unless otherwise stated. Samples formed during this study gelled within 1–2 h [13]; however, samples were left to cure for three days in order to ensure sufficient time for crosslinking to occur. After three days, the jars containing the gels were removed from the oven and left to cool to room temperature. The formed gels were cut into smaller pieces before washing and solvent exchange with acetone (Sigma Aldrich, ≥99.5%). Standard solvent exchange involved addition of ~180 or ~240 mL of acetone to the drained gel, before resealing the lid and, in order to minimise acetone losses, wrapping with paraffin film. Sealed jars were put on a shaker unit (VWR 3500 Analog Orbital Shaker, Lutterworth, UK) and agitated for three days. In the improved solvent exchange method, the exchanged acetone was drained and replaced with 80 mL of fresh solvent on each successive day for three days. After three days of either solvent exchange method, the gel was drained and placed in a vacuum oven (Townson and Mercer 1425 Digital Vacuum Oven, Stretford, UK), preheated to 85 °C (or, in the case of the temperature study samples, the drying temperature was set to match the curing temperature), to dry for two days. Finally, the sample was transferred to a labelled sample tube for storage.

### 4.2. Sample Characterisation

Nitrogen adsorption-desorption measurements were used to obtain textural properties for the RF gel samples prepared in this study. Nitrogen sorption was performed at −196 °C using a Micromeritics ASAP 2420 (Hexton, UK) surface area and porosity analyser. Prior to analysis, samples were outgassed under vacuum below 10 μmHg at 50 °C for 30 min and then at 110 °C for 2 h; except for samples where the influence of temperature was investigated, for these samples, outgassing temperatures matched the gelation and drying temperatures used, and the time for outgassing was adjusted accordingly to ensure removal of all volatile contaminant species. Samples were analysed using a 40 pressure point adsorption and 30 pressure point desorption cycle. All samples were characterised for surface area (m^2^/g), using Brunauer-Emmett-Teller (BET) theory [47], and the Rouquerol correction for microporous samples [42]; total pore volume (cm^3^/g); micropore volume (cm^3^/g) from the t-plot method [48]; and average pore size (nm) from the Barrett-Joyner-Halenda method [49].

Scanning electron microscopy (SEM) micrographs were recorded in backscattered mode at 1000 V using a Field Emission Scanning Electron Microscope (Keysight, U9320B, Wokingham, UK) at magnification 30,000×. Prior to analysis, samples were ground into a fine powder, coated with a 10 nm gold layer using an EM ACE 200 sputter-coater (Leica Inc., Milton Keynes, UK), and attached to aluminium stubs with carbon tape.

## Figures and Tables

**Table 1:** Textural properties of resorcinol–formaldehyde xerogels prepared with standard and improved solvent exchange.

R/C	S_BET_ (m^2^/g)			V_T_ (cm^3^/g)			V_µ_ (cm^3^/g)			$\bar{\varphi }$ (nm)		
	Acetone Exchange Method			Acetone Exchange Method			Acetone Exchange Method			Acetone Exchange Method		
	Standard		Improved	Standard		Improved	Standard		Improved	Standard		Improved
	180 mL	240 mL		180 mL	240 mL		180 mL	240 mL		180 mL	240 mL	
100	480	470	580	0.33	0.33	0.46	0.052	0.046	0.059	3	3	3
200	470	530	500	0.54	0.71	0.73	0.040	0.056	0.052	5	5	6
300	420	430	470	0.78	0.93	1.05	0.043	0.052	0.060	8	10	11
400	370	300	220	0.97	0.95	0.99	0.046	0.035	0.033	12	14	24
500	300	220	230	0.97	0.96	1.17	0.039	0.033	0.034	16	24	29
600	230	110	220	1.01	0.44	0.81	0.036	0.019	0.036	24	27	22

S_BET_—surface area from Brunauer-Emmett-Teller (BET) analysis; V_T_—total pore volume determined from adsorption at p/p° ~1; V_µ_—micropore volume determined using t-plot method; $\bar{\varphi }$—average pore width from Barrett-Joyner-Halenda (BJH) analysis. Errors are omitted from the table as all values are reported to an accuracy less than the largest error for each variable.

**Table 2:** Textural properties of resorcinol–formaldehyde xerogels prepared using different percentage solids contents.

*w*/*v*% Solids	S_BET_ (m^2^/g)			V_T_ (cm^3^/g)			V_µ_ (cm^3^/g)			$\bar{\varphi }$ (nm)		
	R/C Ratio			R/C Ratio			R/C Ratio			R/C Ratio		
	100	300	600	100	300	600	100	300	600	100	300	600
10	500	370	-	0.36	0.85	-	0.057	0.037	-	3	9	-
20	500	490	280	0.32	0.91	1.00	0.065	0.064	0.046	3	8	18
25	550	410	190	0.42	1.00	1.07	0.054	0.042	0.030	3	10	32
30	570	490	260	0.46	1.08	1.17	0.055	0.064	0.045	3	9	28
35	570	450	260	0.45	0.98	1.23	0.051	0.050	0.038	3	9	27
40	540	550	330	0.44	1.07	1.53	0.048	0.077	0.056	3	9	29

S_BET_—surface area from BET analysis; V_T_—total pore volume determined from adsorption at p/p° ~1; V_µ_—micropore volume determined using t-plot method; $\bar{\varphi }$—average pore width from BJH analysis. Errors are omitted from the table as all values are reported to an accuracy less than the largest error for each variable.

**Table 3:** Textural properties of resorcinol–formaldehyde gels dried at ambient pressure and under vacuum.

R/C	S_BET_ (m^2^/g)		V_T_ (cm^3^/g)		V_µ_ (cm^3^/g)		$\bar{\varphi }$ (nm)	
	Drying Method		Drying Method		Drying Method		Drying Method	
	Ambient	Vacuum	Ambient	Vacuum	Ambient	Vacuum	Ambient	Vacuum
100	510	600	0.45	0.47	0.037	0.064	4	3
300	380	460	1.11	1.12	0.044	0.064	13	12
600	90	120	0.31	0.54	0.014	0.023	19	30

S_BET_—surface area from BET analysis; V_T_—total pore volume determined from adsorption at p/p° ~1; V_µ_—micropore volume determined using t-plot method; $\bar{\varphi }$—average pore width from BJH analysis. Errors are omitted from the table as all values are reported to an accuracy less than the largest error for each variable.

**Table 4:** Textural properties of resorcinol–formaldehyde xerogels prepared at different temperatures.

T (°C)	S_BET_ (m^2^/g)			V_T_ (cm^3^/g)			V_µ_ (cm^3^/g)			$\bar{\varphi }$ (nm)		
	R/C Ratio			R/C Ratio			R/C Ratio			R/C Ratio		
	100	300	600	100	300	600	100	300	600	100	300	600
45	-	<1	20	-	-	0.07	-	-	0.002	-	-	9
55	<1	140	100	-	0.14	0.48	-	0.010	0.011	-	4	22
65	370	350	200	0.22	0.52	0.82	0.054	0.036	0.027	3	6	20
75	530	440	220	0.37	0.77	0.82	0.064	0.052	0.030	3	8	21
85	580	470	220	0.46	1.05	0.81	0.059	0.060	0.036	3	11	22
95	610	490	230	0.52	1.18	0.92	0.057	0.064	0.038	4	12	24

S_BET_—surface area from BET analysis; V_T_—total pore volume determined from adsorption at p/p° ~1; V_µ_—micropore volume determined using t-plot method; $\bar{\varphi }$—average pore width from BJH analysis. Errors are omitted from the table as all values are reported to an accuracy less than the largest error for each variable.
